# 

**Published:** 1843-06

**Authors:** 


### CONTENTS

OF NO. VI.

### ORIGINAL COMMUNICATIONS.

### ESSAYS AND CASES.

Art. I.—St. Louis Hospital Reports. No. 1. By M. L. Linton, M. D., Professor of Obstetrics in the Medical Department of the University of St. Louis. - 401

Art. II.—Amputation of the Right Arm at the Shoulder Joint. By T. A. Tellkampf, M. D., of Cincinnati, Ohio. 405

Art. III.—Cases of Tardy Fecundity.—Translated from a French manuscript for the Western Journal of Medicine and Surgery. By Daniel Stahl, M. D., of Vincennes, Indiana. - - - - - 408

Art. IV.—Congestive Fever. By M. M. Pallen, M. D., of St. Louis, Mo. - - - - - - 412

Art. V.—A Case of Pneumonia produced by Asphyxia. By G. W. Bayless, M. D., Demonstrator of Anatomy in the Louisville Medical Institute. - - 422

### BIBLIOGRAPHICAL NOTICES.

Art. VI.—A Practical Treatise on Venereal Diseases; or Critical and Experimental Researches on Inoculation applied to the Study of those Affections, with a Therapeutical Summary and Special Formulary. By Ph. Ricord, M. D., Surgeon of the Venereal Hospital of Paris, Clinical Professor of Special Pathology, &c. Translated from the French by Henry Pilkington Drummond, M. D. Philadelphia, Lea and Blanchard: 1843. pp. 256. - - - - - 426

Art. VII.—A Treatise on Diseases of the Eye. By William Lawrence, F. R. S., Surgeon Extraordinary to the Queen, Surgeon to St. Rartholomew's Hospital, &c.,

&c. From the last London edition. With numerous additions and sixty-seven Illustrations. By Isaac Hays, M. D., Surgeon to Will's Hospital, &c., &c. 427
Art. VIII.—A Treatise on Ruptures. By W. Lawrence, F. R. S., Surgeon extraordinary to the Queen, &c. From the fifth London edition. Revised, corrected and enlarged, pp. 380, 8vo. Lea and Blanchard; Philadelphia, 1843. - - - - - 428
Art. IX.—Proceedings of the Medical Convention of Ohio, held at Cincinnati on the 16th, 17th, 18th, 19th, and 20th, of May, 1842. With selections from papers read before that body. Cincinnnati, R. P. Brooks, 1842. pp. 51. - - - - - - 429

SELECTIONS FROM AMERICAN AND FOREIGN JOURNALS.

Presumption of Survivorship. - - - - - 442
A tabular view of the treatment of Uterine Hemorrhages. By William Camps, M. D., Edin. - - - 452
On Primary Syphilitic Buboes. By De Castlenau. - 456
On the Causes and Treatment of Goitre. - - - 456
Asthenic Amaurosis cured by the use of convex Spectacles. - 457
Structure of the Arteries. - - - - - - 458
On Fatty Degeneration of the Arteries, with a Note on some other Fatty Degenerations. By George Gulliver, F. R. S. 460
Introduction of Air in the Veins. - - - - - 460
Dry Feet. - - - - - - - - 461
Effects of Arsenic upon Sheep. - - - - - 462
Employment of Muriate of Ammonia in Scirrhus of the Stomach. 463
The Physicians of Dantzic Sixty Years Since. - - 464
Improved Treatment of Hydrocele. - - - 464
Spermatozoa Observed a Second time within the Ovum. - 465
A Colony of Seven Hundred Lunatics. - - - 465
Researches on the Blood. - - - - - - 466
Vaccine lymph from the pustules produced by tartarized Antimony. - - - - - - - 466
Renal disease Simulating Calculus in the Bladder. - 467
Electro-Puncture. - - - - - - - 467
The Rage for Invention. - - - - - - 468

ORIGINAL INTELLIGENCE.

Travelling Editorials. - - - - - - 469
New Work on Southern diseases. - - - - - 475
Army Medical Board. - - - - - - 476
St. Louis Hospital Reports.—St. Louis Medical and Surgi- Journal. - - - - - - - 476
Medical Journal in Havana, - - - - - 476
Index. - - - - - - - - - 477

